# Glycogen and Glycosylation: Friends or Foes?

**DOI:** 10.3390/biom16060885

**Published:** 2026-06-16

**Authors:** Rohit Sai Reddy Konada, James Osborn, Sharmistha Mitra

**Affiliations:** 1 Department of Pediatrics, Division of Neurology, UT Southwestern Medical Center, Dallas, TX 75390, USAjames.osborn@utsouthwestern.edu (J.O.); 2 Department of Biochemistry, UT Southwestern Medical Center, Dallas, TX 75390, USA

**Keywords:** glycogen, glycans, N-linked, O-linked, glucose, ubiquitin, polyglucosan body, LUBAC

## Abstract

Glycosylation, glycogen metabolism, and ubiquitination represent three fundamental cellular processes that are traditionally studied as distinct aspects of biology. Glycosylation and glycogen metabolism are unique carbohydrate-based pathways. The process of glycosylation generates structurally diverse glycans that regulate protein folding, cell signaling, and host–pathogen interactions, while glycogen serves as a glucose reserve essential for energy homeostasis. Emerging evidence reveals a deep mechanistic connection between these pathways, particularly in the context of brain biology and inherited metabolic diseases. Here, we present recent research linking glycosylation defects with glycogen metabolism, highlighting how changes in the shared metabolites and enzymatic pathways contribute to human health and disease. We then discuss the overlapping disease symptoms of congenital disorders of glycosylation and glycogen storage diseases, with particular emphasis on polyglucosan body-forming diseases. We also highlight the role of non-canonical ubiquitin ligase complexes such as laforin–malin and LUBAC and present emerging evidence for their potential role in the glycogen quality-control mechanism. Finally, we review current therapeutic strategies for CDGs and GSDs, including monosaccharide supplementation, glycogen synthase modulation, and gene therapy. Together, this review underscores glycogen as more than an energy store—as a key contributor to glycosylation homeostasis and cellular regulation in health and disease.

## 1. Introduction

Glycomics explores glycan biosynthesis, structure, and functions in organisms. Glycans are carbohydrate chains covalently linked to lipids and proteins through the cellular process of glycosylation [1,2]. Protein glycosylation is a crucial post-translational modification in health and disease where proteins can be glycosylated at either asparagine or serine/threonine residues to form N-linked and O-linked glycoproteins [3]. Being diverse, dynamic, and ubiquitous in all forms of life, glycans are implicated in many biological roles. Hence, glycans are categorized by their structural function, inter- and intraspecific recognition roles, and molecular mimicry [4]. Structurally, glycosylation could serve as a scaffold that regulates protein folding and maintains physical integrity [5]. In host–pathogen interactions, pathogens and their toxins bind specific host glycans to facilitate infection. Additionally, some of the host glycans act as decoy receptors to defend against pathogenic effector intoxication [6]. Intrinsically, glycans mediate cell signaling and immune regulation through self-recognition [4].

Most glycoconjugates are localized on the cell surface, providing a vantage point that allows them to interact dynamically with the external environment and perform their critical biological functions. To date, in animals, nine common monosaccharides have shown to contribute to the structural complexity of glycans through variations in composition, branching patterns, and specific linkage types. In addition to these variables, terminal monosaccharides can be further modified by fucosylation (the enzymatic addition of fucose), sialylation (the transfer of sialic acid residues), and sulfation (the addition of sulfate groups), exponentially increasing their structural diversity [7]. While traditionally glycans were known to conjugate with proteins and lipids, a recent study has identified glycosylated RNA on cell surfaces, suggesting exciting possibilities of glycoconjugates forming between glycans and other non-proteinaceous macromolecules [8].

The process of glycosylation occurs within the Endoplasmic Reticulum (ER)–Golgi network through the sequential action of glycosyltransferases and glycosidases [9]. Given the critical importance of this cellular process, genetic defects in these enzymes result in severe, often lethal conditions categorized as Congenital Disorders of Glycosylation (CDG) [10]. Historically, the study of CDGs has been instrumental in advancing our understanding of the glycosylation pathways.

The structural diversity and complexity of the glycome results in significant genotypic and phenotypic heterogeneity among CDGs, posing a substantial challenge for clinical diagnosis [11]. Most common manifestations of CDG include developmental delay, facial dysmorphisms, and cardiac and neurological abnormalities. Intriguingly, apart from CDGs, glycosylation defects have recently been associated with a distinct metabolic group of diseases called glycogen storage disorders (GSDs) [12]. This group of glyco-metabolic diseases are caused by faulty enzymes involved in the biosynthesis, degradation, structural integrity, and solubility of the glycogen macromolecule. These disorders are congenital, occurring in all age groups, and often impact multiple organs such as the liver, skeletal muscles, heart, and brain.

In this review, we surveyed recent research linking glycosylation to glycogen biology; we then introduce the role of ubiquitination (another major post-translational modification) in the above two biological processes. We further explore how glycosylation depends on ubiquitination, as seen through the lens of glycogen metabolism.

## 2. Glycosylation: The Process and Associated Diseases

**Figure 1:**
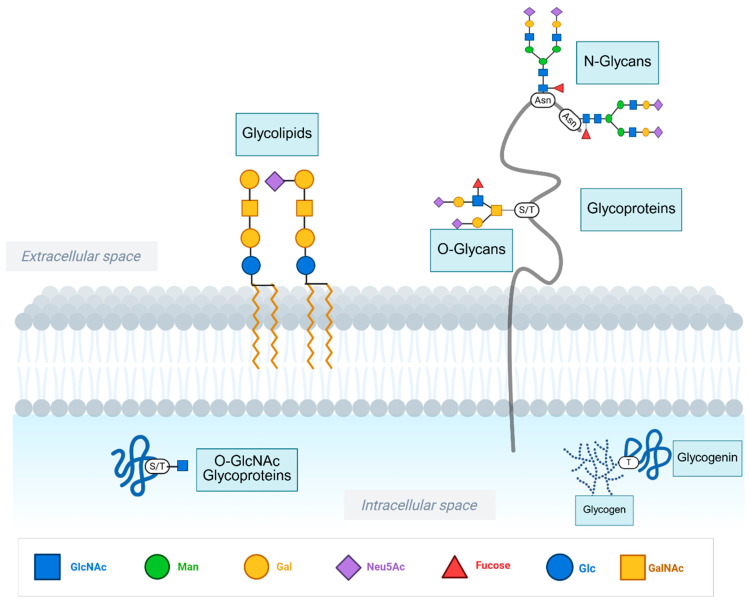
Important glycoconjugates of mammalian cells. Glycoconjugates are formed when sugar moieties are attached to either proteins or lipids. N-Glycans, through GlcNAc, are attached to the asparagine residues of the glycoproteins and O-Glycans, through GalNAc, are attached to the serine/threonine residues. Glycolipids contain sialo or asialo glycans attached to the ceramide moiety of the sphingolipids. Many nuclear and cytoplasmic proteins contain a single O-GlcNAc attached to serine/threonine residue. Glycogen is also a type of glycoconjugate where glucose units are attached to the tyrosine residue of the glycogenin protein. Created withCreated in BioRender. Mitra, S. (2026) https://BioRender.com/3p4mpi0 (accessed on 7 May 2026).

Glycoproteins and glycolipids are the major glycoconjugates present in mammals. The glycosylation of proteins is a major post-translational modification and more than 50% of proteins are glycosylated in the cells [13]. Two types of glycoproteins are present based on the sidechain of the amino acid of the protein conjugated to the glycans: N-linked and O-linked glycoproteins (Figure 1). In N-linked glycoproteins, the glycans are attached to the amino acid group sidechain of asparagine (Asn). This is the most common protein post-translational modification in eukaryotes and involves the assembly and trimming of the attached oligosaccharide in the cytosol ER and Golgi complex [14]. N-glycosylation is initiated by the transfer of N-acetylglucosamine (GlcNAc) phosphate from the UDP-GlcNAc to ER membrane-bound dolichol monophosphate (Dol-P) on the cytosolic face, forming GlcNAc-pyrophosphatedolichol (GlcNAc-PP-Dol). Subsequent addition of one GlcNAc and five mannose (Man) results in Dol-PP-heptasaccharide (Man5GlcNAc2). At this stage, the lipid-linked heptasaccharide is translocated into the lumen side of the ER membrane by the enzyme flippase, allowing the resident mannosyltransferases and glucosyltransferases to sequentially add four Man and three Glucose (Glc) residues to generate Glc_3_Man_9_GlcNAc_2_-P-P-Dol. This mature N-glycan precursor is transferred to Asn residues in proteins with Asn-X-Ser/Thr sequence via oligosaccharyltransferase (OST). Once transferred, the precursor N-glycan is further trimmed by the ER-glucosidases (glucosidases I, II, and alpha-mannosidase I). The processed glycan targets the glycoprotein to the Golgi complex where further maturation of the N-glycan is accomplished [15,16,17,18,19]. A minor portion of the ER-glycoproteins (hydrolases) are tagged by mannose-6-phosphate through the action of GlcNAc-phosphotransferase. This tagging helps in the localization of hydrolases to lysosomes and helps in lysosomal functioning [20].

In contrast to N-glycosylation, O-glycans are attached to the hydroxyl group sidechains of serine and threonine amino acids of proteins. Unlike N-glycosylation, the process of O-glycosylation occurs entirely in the Golgi and contains only the assembly and not the trimming part [21].

The defects in either N- or O-glycan moiety synthesis, or their attachment to the protein and lipids, cause CDG [22]. CDG constitutes about 160 diseases affecting the synthesis of glycoproteins, glycolipids, and other glycosylation forms [1]. In general, CDGs are autosomal recessive with few dominant and X-linked disorders. CDGs are heterogeneous, multisystemic disorders categorized into four groups based on the glycosylation pathway affected (N-linked, O-linked, glycolipids, or multiple glycosylation pathways affected) [23]. CDGs are rare due to embryonic lethality, and patients that survive are hypomorphic with residual activities of the glycosylation pathways. This highlights the critical roles of glycans in human biology. The nervous system is affected in more than 80% of the CDGs identified [24]. Defects in the N-glycosylation process are shown to exhibit aberrant neurological effects. In fact, out of the fifteen N-glycan CDGs, fourteen are known to show nervous system defects, emphasizing the important role of N-glycans in brain development and function. As an example, Phosphomannomutase 2-congenital disorder of glycosylation (PMM2-CDG), the most common glycosylation disorder identified with more than 1000 cases worldwide, presents with severe developmental and motor defects with dysmorphic features [25]. This emphasizes the importance and involvement of the central nervous system in CDGs.

Intriguingly, this connection between brain biology and glycosylation is also accentuated by a recent discovery, which identified brain glycogen as an important source of glucosamine, which is essential for the synthesis of monosaccharides for N-linked protein glycosylation [26]. However, to understand any potential connection between brain glycogen, glycosylation, and their implication in human diseases, we will need to understand glycogen and its metabolism, which we describe in the next section.

## 3. Glycogen and Its Metabolism

In the context of glycobiology, glycogen is the less studied macromolecule compared to glycans. Glycogen is a glucose polymer present in all heterotrophic organisms. It is the largest cytoplasmic molecule and a major storage of polysaccharides in animals. However, it also plays a central role in short-term storage and energy release in certain bacterial species [27]. Liver and skeletal muscles are the major storage sites of glycogen. Glycogen in the brain has many crucial roles. Astrocytic glycogen accounts for the majority of glycogen in the brain but neuronal cells also possess glycogen stores [28]. Glycogen is found in higher amounts in the surface levels of the cortex, hippocampus, the thalamic reticular nuclei, and the molecular layer of the cerebellum, and is generally found in higher amounts in areas with higher neural activity [29].

The brain alone accounts for almost 20 percent of the body’s total energy usage [30]. During a lapse in this energy supply, brain glycogen stores are catabolized instead. However, the high energy demand leads to rapid depletion of these reserves if the normal energy supply is not reestablished [31]. The glycogen reserves are crucial to maintain normal brain function during lapses of energy and hypoxic conditions [32]. Disruption of these brain glycogen reserves has a downstream effect, leading to various lethal neurological diseases. Abnormal glycogen storage in the brain can lead to rare but lethal neurological diseases categorized by insoluble glycogen deposits, such as seen in Lafora disease and Adult Polyglucosan Body Disease [30,33]. The lethality of these diseases highlights the crucial role glycogen plays in normal brain function. Additionally, glycogen metabolism is necessary for glutamatergic neurotransmission and excitatory neurotransmission in general. It acts as a supply for glutamate production and is coupled to the removal of extracellular K^+^ ions by astrocytes [31].

**Figure 2:**
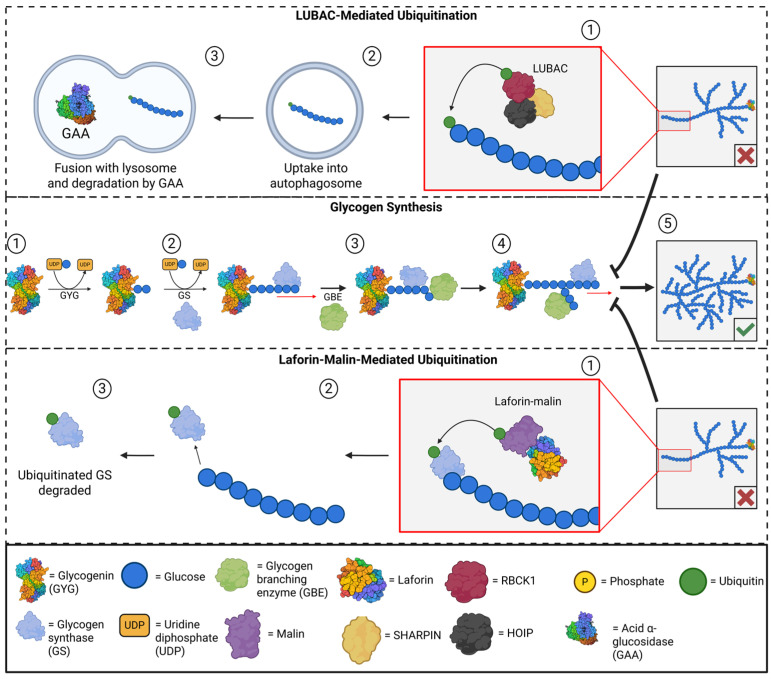
Glycogen synthesis, LUBAC, and laforin–malin-mediated ubiquitination. Glycogen synthesis: new glucose monomers are donated via uridine diphosphate (UDP). (1) Glycogenin (GYG) primes chain synthesis via auto-glucosylation. (2) Chain is further elongated via glycogen synthase (GS) with α-1,4-linkages. (3) Glycogen branching enzyme (GBE) periodically introduces branching points to the chain via α-1,6-linkages. (4) GS further elongates both parent chain and branched chain. (5) The GS/GBE action results in the formation of glycogen’s macromolecular structure. Laforin–malin–mediated degradation: (1) Laforin–malin ubiquitinates GS on overly long chains that is formed due to overactive GS. (2) GS–Ubiquitin releases from the overly long chain, preventing further elongation. (3) GS–Ubiquitin is degraded. LUBAC-mediated degradation: (1) RBCK1, in complex with HOIP and SHARPIN, monoubiquitinates overly long chain to signal for degradation. (2) Glycogen chain is encapsulated in autophagosome. (3) Autophagosome fuses with lysosome (autolysosome), allowing for degradation by acid α-glucosidase (GAA). Created with Created in BioRender. Mitra, S. (2026) https://BioRender.com/3wguep1 (accessed on 7 May 2026).

Structurally, glycogen is a homopolymer of glucose and is synthesized in the cytoplasm through a cellular process called glycogenesis. Glycogen synthesis or glycogenesis is a multi-step process regulated by three key proteins: glycogenin (GYG), glycogen synthase (GS), and glycogen branching enzyme (GBE) [34]. GYG initiates biosynthesis by transferring glucose from the nucleotide-activated sugar donor UDP-glucose to one of its tyrosine residues. This autoglucosylation process forms a linear glucan structure comprising around 20 glucose monomers linked through α1-4 glycosidic bonds. The elongation enzyme GS catalyzes the addition of more glucose residues to the nascent glucan chain via α1-4 linkages at the non-reducing end. In coordination with GS, the branching enzyme GBE transfers six to eight glucose units to the non-reducing end via α1-6 glycosidic bonds. This orchestrated catalysis by these two glucosyltransferases results in a soluble, globular, and branched glycogen polymer readily available for cellular metabolism (Figure 2). Glycogen also contains phosphate in trace amounts along with the glucose units; however, the kinase protein responsible for placing this phosphate onto glycogen molecules is unknown. In some studies, it is proposed that glycogen synthase during its catalysis reaction may incorporate the phosphate onto glycogen in an error [35].

The catabolism of glycogen occurs In both cytoplasm and lysosomes [36]. Cytoplasmic degradation of glycogen, termed as glycogenolysis, is catalyzed by two enzymes: glycogen phosphorylase (GP) and glycogen debranching enzyme (GDE). GP cleaves the α1-4 glycosidic bonds from the non-reducing ends of the linear chains by releasing glucose-1-phosphate. GP catalytic activity halts at four glucose residues away from the branch point. At this stage, the bi-functional enzyme GDE first transfers the three glucose residues to another non-reducing end linear chain by its transferase activity. Using glycosidase activity, GDE cleaves the α1-6 glycosidic bond at the branch point, releasing free glucose. A second catabolic pathway for glycogen degradation occurs in the lysosomes through the process called glycophagy [37]. This phagic-mediated glycogenolysis involves bulk degradation of glycogen through α-glucosidase (GAA) enzyme. Unlike the cytoplasmic glycogenolytic enzymes GP and GDE, GAA hydrolyses both α1-4 linkage, and the branched 1-6 linkages.

Apart from synthesis and degradation, recent research from our group and others has shed light on additional non-canonical enzymes of glycogen metabolism, which are responsible for maintaining the structural integrity of glycogen. Laforin is a glycogen phosphatase and regulates the levels of phosphate groups in glycogen [38]. Malin is an E3 ubiquitin ligase. In complex with laforin, it helps in the regulation of key glycogen metabolism-related enzymes [39,40] (Figure 2). It is the loss of function of either laforin or malin that results in polyglucosan body formation in the brain, muscle, heart, and liver specifically known as Lafora bodies [41,42,43]. In addition to laforin–malin, there is another E3-ligase complex named Linear ubiquitin chain assembly complex (LUBAC) which is involved with glycogen metabolism [44,45,46]. The mechanism behind LUBAC’s control over glycogen metabolism is unknown but is hypothesized to be recruited to unbranched glycogen to facilitate its degradation [47] (Figure 2). LUBAC is a heterotrimeric complex of two RBR-type E3 ligases, heme-oxidized IRP2 ubiquitin ligase 1 (HOIL-1L; RBCK1) and HOIL-1L interacting protein (HOIP), and the adaptor protein SHANK-associated RH domain-interacting protein (SHARPIN). As the name suggests, LUBAC plays a key role in the ubiquitinating, post-translational modification of proteins [44]. Specifically, LUBAC is involved in regulating key processes such as TNF signaling, redox homeostasis, and TLR signaling [48,49]. In addition to these roles, emerging research shows a crucial role in proper glycogen storage. Clinical findings in patients with RBCK1 deficiency show polyglucosan body accumulation in tissue, leading to cardiac and skeletal myopathy [50]. This reinforces LUBAC’s involvement in the glycogen metabolic process. While the underlying mechanisms have yet to be fully uncovered, it has been demonstrated in vitro that RBCK1 will ubiquitinate glycogen and maltoheptaose, an analog for over-elongated glycogen chains, in an oxyester-linked manner.

## 4. Glycogen and Glycosylation

Glycogen is the carbohydrate reserve that supplies glucose for the cell’s metabolic needs. Liver glycogen is known to maintain blood glucose in starvation, and skeletal glycogen mobilizes glucose for muscle contraction. The function of glycogen in other tissues, like the brain, is poorly understood [36]. Glucose is the key monosaccharide in carbohydrate metabolism. The glycogenolysis pathway produces glucose-1-phosphate, a key intermediate for either direct energy generation by glycolysis, or it can be used to synthesize nucleotide-activated sugar UDP-glucose [51]. These nucleotide sugars are used as donor substrates by glycosyltransferases for glycosylation reactions [51]. The UDP-Glc pool is used in the synthesis of glucosylceramide and dolicol-P-glucose, the important precursors for the N-glycan biosynthetic pathway [52]. The epimerization of UDP-Glc to UDP-Galactose by UDP-Gal4-epimerase enzymes provides the sugar donor for the galactosyltranserases, enabling them to synthesize N-linked glycans, O-linked glycans, and glycolipids [52].

All above emphasize the role of glycogen in cellular glycosylation pathways. In a more recent study, in addition to glucose, 25% of brain glycogen was shown to be composed of glucosamine, the precursor for the generation of UDP-GlcNAc, and is required for the biosynthesis of N-glycans and glycolipids [26]. Glycogen and glycosylation have intertwined roles in brain function. Glycogenolysis is utilized in the synthesis of UDP-GlcNAc for N-glycosylation. Glucosamine is attached to glycogen, which then acts as a reservoir for glucosamine metabolism and the synthesis of UDP-GlcNAc [27]. In addition to biochemical studies, localization studies have revealed the close association of glycogen molecules with the cytoplasmic side of ER [53,54]. It was proposed that this close association is required for the rapid channeling of metabolic substrates required for ER functions. The association of glycogen with ER may also have consequences for protein glycosylation because, as described before, initial important steps of the process occur in ER. The vital polysaccharides of the cell, glycogen and glycans, share similar donor substrates: UDP-Glc and UDP-GlcNAc. Glycogen synthase (GS) utilizes UDP-Glc to synthesize glycogen through the process of glycogenesis in the cytoplasm. As mentioned above, recent research has shown that GS also utilizes UDP-GlcNAc to incorporate glucosamine in the brain glycogen. On the other hand, glycosyltransferases residing in ER and Golgi utilize the UDP-Glc/GlcNAc along with other nucleotide sugars to synthesize complex glycans present on glycolipids and glycoproteins. Even though these two crucial metabolic processes are separated compartmentally, the substrates can shuffle between these organelles using membrane transporters. The metabolic enzymes of glycogenesis and glycosylation compete for the donor substrates, which establishes a tight regulatory homeostatic flux between them.

As described in the introduction, GSDs result from the defects in the synthesis and degradation of the glycogen molecule or even due to failure in maintaining its structure and solubility. Therefore, GSD patients suffer from abnormal glycogen storage where the quantity or the quality of the glycogen molecule is affected. In certain types of GSDs, where the quality of glycogen is affected, accumulation of abnormally structured glycogen leads to polyglucosan body (PB) formation [55,56].

PBs are insoluble glycogen aggregates that are toxic to cells, often leading to neurodegeneration (brain accumulation), cardiomyopathy (heart accumulation), and skeletal myopathy (muscle accumulation). Adult polyglucosan body disease (APBD), due to GBE1 deficiency (GSD IV), Lafora Disease (deficiency of either laforin or malin), Poluglucosan Body Myopathy 1 (PGBM1) due to HOIL-1L-deficiency, and Polyglucosan Body Myopathy 2 (GSD XV, due to deficiency in Glycogenin 1 enzyme) are examples of PB-GSDs.

Clinically, GSDs exhibit phenotypic continuum and are based on the enzyme affected. They are categorized from GSD0 through XV [57]. GSD 0b, resulting from glycogen synthase 1 deficiency, is characterized by cardiomyopathy, arrythmia, general muscle weakness, and increased risk of cardiac arrest in the patient’s lifetime. GSD II (Pompe disease) results from Gaa deficiency with skeletal and cardiac muscle weakness, and respiratory issues with lysosomal accumulation of soluble glycogen molecules in multiple organs. GSD XV (Polyglucosan Body Myopathy 2) resulting from GyG deficiency is characterized by skeletal and cardiac myopathy and general muscle weakness. What is intriguing is that clinical data obtained from some GSD patients (type Ib, III, IV, and XIV) showed abnormal glycosylation as well [26,58,59,60]. Studies on mouse models of neurological GSD III and Lafora disease revealed that brain PGBs trapped glucosamine monosaccharides, the building blocks of UDP-GlcNAc, which are required for the synthesis of N-glycans [26]. In fact, some GSDs are now classified as CDGs; for example, GSD XIV (phosphoglucomutase 1 deficiency) patients, along with PGB accumulation, exhibit protein hypoglycosylation. Table 1 lists the GSDs that also show glycosylation defects.

Finally, in an intriguing possibility, it was proposed that glycogen, especially long glucan chain-containing glycogen molecules, could be ubiquitinated by LUBAC and taken to lysosome for its degradation before it amasses into PBs [47]. In unusual cases, ubiquitination is known to happen to non-canonical molecules such as GlcNAc attached to proteins influencing their cellular function. SKP1-CUL1-F-box (SCF)^FBS2/FBXO6^ is an E3 ubiquitin ligase that ubiquitinates N-GlcNAc residues onto Nuclear Respiratory Factor 1 (Nrf1) transcription factor. This ubiquitination ultimately inhibits Nrf1 activation [62]. Recently, it was also shown that tissue glycogen, especially from liver, is heavily ubiquitinated [63]. It is known that glucosamine is present in liver glycogen [64]. Whether GlcNAc present on glycogen could also be ubiquitinated needs to be investigated in the future.

Taken together, the clinical findings from GSDs, the overlapping metabolite profiles, and the subcellular localization patterns provide compelling evidence for a deep mechanistic connection between glycogen metabolism and glycosylation pathways.

## 5. Box 1: Connection Between GSDs and Ubiquitination

Ubiquitination is another major post-translational modification in cells where a small 8 kDda protein, ubiquitin, is attached to another protein or other macromolecules. This addition ends up regulating the substrate’s stability, activity, and localization [65]. The process of ubiquitination is a sequential biochemical process that involves the enzymatic actions of three enzymes (E1, E2, and E3 ubiquitin ligases) that, in the last step, take the ubiquitin from E2 and attaches it onto the targets. The canonical targets for this ubiquitination process are proteins of the cell; however, recent research has shown that non-canonical targets for ubiquitination could include diverse molecules such as phospholipids, lipopolysaccharides, glycans, ribose sugars, and glycogen [66].

A direct connection between glycogen and the ubiquitination process was reinforced by the discovery of certain GSDs due to recessive mutations in E3 ligase enzymes [67]. Lafora Disease is a fatal form of epilepsy in teenage children due to mutations in either the EPM2A or EPM2B genes. The EPM2B gene encodes malin E3 enzyme that interacts with EPM2A-encoded laforin phosphatase, ubiquitinating glycogen metabolic enzymes and controlling glycogen solubility in an unknown pathway [42,67]. LUBAC is another ubiquitin ligase complex composed of two E3 ligases—RBCK1 (also known as HOIL-1L) and HOIP—and an adaptor protein SHARPIN. Mutations in either RBCK1 or HOIP lead to severe cardio and skeletal myopathy in humans, with insoluble glycogen accumulated in different organs [68,69]. KLHL24 protein is part of a Cullin-RING E3 ubiquitin ligase complex, and mutation in this protein leads to PB accumulation and fatal cardiomyopathy as well [70]. Finally, the IRF2BPL (Interferon Regulatory Factor 2 Binding Protein-Like) gene encodes for the IRF2BPL protein that acts as both a transcription factor as well as an E3-ubiquitin ligase [71]. In some patients, mutations within the IRF2BPL gene drive soluble glycogen to become insoluble and accumulate in sweat glands [71]. The exact pathway by which each of these E3 enzymes control glycogen solubility is yet to be discovered. However, a recent paper describing tissue glycogen ubiquitination for the first time [63] has raised the possibility of non-canonical glycogen ubiquitination by one or more of these E3 ligases that may be central in glycogen solubility. Hence, in the manifestation of GSDs, the functionality of these enzymes are lost.

## 6. New Treatment Regime for CDGs from GSD Perspective

Both CDGs and GSDs share a fundamental aspect of being heterogenous, mostly pediatric genetic disorders, characterized by deficient glycosylation and glycogen metabolism, respectively [12,23]. Being rare, and biochemically and clinically diverse, most CDGs have only been identified within the last two decades. With the advancement of glycan analytical methods, new subtypes of CDGs have been identified in recent years [72]. Although advances in analytical methods have enabled more accurate identification of CDGs, therapeutic development remains limited due to the small number of affected individuals [73]. Currently, established therapies for CDGs include supplementation of monosaccharides and cofactors, delivery of enzymes, monosaccharide precursors, and chaperons [74,75]. Mannose, galactose, and fucose supplementations are classic examples for treating CDGs [76,77]. MPI-CDG is caused by a deficiency of the MPI (mannose-6-phosphate isomerase) enzyme. The resulting patients cannot convert fructose-6-phosphate to mannose-6-phosphate, which is essential for GDP-mannose synthesis required for N-glycosylation. Oral supplementation of mannose improved clinical and biochemical conditions of both MPI-CDG and PMM2-CDG patients [78,79]. Similarly, galactose supplementation improved the symptoms of SLC35A2-CDG patients [80]. SLC35A2 is the UDP-galactose transporter residing on the Golgi membrane and the deficiency of this transporter impairs the activity of galactosyl transferases required for all the types of glycosylation pathways [80].

For GSDs resulting in hyperaccumulation of PBs, strategies to downregulate GS or degrade already existing PBs have been successful. In this context, downregulation of GS mRNA via antisense oligonucleotides, the CRISPR-Cas9 system, shRNA, or by miRNA has shown promise in preclinical models of multiple GSDs [81,82,83,84]. Reduction in GS through small molecules and inhibitory compounds has also been shown to decrease glycogen concentrations [85,86]. Two recent studies in Lafora disease (LD) show therapy targeting the rescue of laforin or malin as a potential avenue for human disease cure. Mice with either laforin or malin deficiency were rescued from neuropathy and PB formation with intravenous delivery of rAAV2/9P31 viral vectors with the corresponding laforin or malin gene, as well as an AAV9-mediated therapy rescuing malin-deficient mice from LD with no toxic effects [87,88]. Gene therapies for other GSDs are also being actively studied [57]. GSD Ia, resulting from a deficiency in glucose-6-phosphatase α, has a proposed AAV8-mediated therapy targeting glucose-6-phosphatase catalytic subunit (G6PC) rescue that has advanced to an active Phase III double-blind placebo clinical trial (NCT05139316) [89]. Pompe disease (GSD II) resulting from GAA deficiency has one active Phase I (NCT05567627) and two active Phase I/II clinical trials (NCT04174105, NCT04093349) for AAV vectors targeting rescue of GAA activity [90,91,92]. Enzyme replacement therapy (ERT) shows promise for Pompe disease, with three ERT treatments approved for use in the US: Myozyme (alglucosidase alfa), Nexviazyme (avalglucosidase alfa), and a combination of Pombiliti and Opfolda (cipaglucosidase alfa and miglustat, respectively) [93,94]. This ERT involves intravenous delivery of recombinant human GAA, conjugated with mannose-6-phosphate moieties to facilitate its uptake into the cell and lysosome, where it can rescue the function of GAA. Finally, reduction in PBs has been shown in mice to be an effective method of reducing LD symptoms; thus, therapies targeting delivery of glycogen degradation enzymes to the cell are a potential treatment strategy for LD [95]. Antibody-enzyme fusion (AEF) therapy involves antibody conjugation of these enzymes to facilitate delivery to the necessary intercellular compartments. Antibody conjugation allows for binding and intercellular delivery of enzyme therapy. AEF-mediated delivery of rhGAA (VAL-1221) and pancreatic α-amylase (VAL-0417) via intracerebroventricular (ICV), intramuscular, or tail vein injection drastically reduced glycogen levels in both laforin and malin KO mice [96]. Table 2 lists the examples of some of the current therapeutic strategies available for both CDGs and GSDs.

In cases where the phenotypic spectrum of CDG and GSD overlaps (see Table 1 for the list of such diseases), a new regime for CDG treatment can be optimized by exploiting available GSD therapeutics (Table 2). In fact, a study applied such an approach for LD where they utilized an antibody-enzyme fusion with α-amylase activity to selectively hydrolyze PBs in brain [26]. MALDI glycogen and glycan imaging revealed the rescue of hypoglycosylation in the brain of LD mice, strongly suggesting that GSD therapeutics can rescue CDG symptoms. As glucosamine is now an established part of glycogen, which is incorporated by active GS, future therapies to reduce GS activity by the methods discussed above such asactivating polyglucosan degradation using enzyme replacement or bringing back glycogen metabolism-related genes by gene therapies may also rescue glycosylation defects observed in overlapping CDGs.

## 7. Conclusions and Perspectives

**Figure 3:**
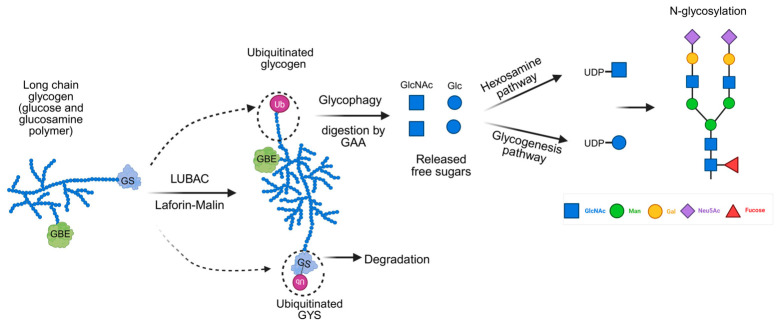
Schematic of the interplay between glycogen metabolism, glycosylation, and ubiquitination. Glycogen synthase (GS) catalyzes the synthesis of polyglucans with interspersed glucosamine residues, while glycogen branching enzyme (GBE) adds α-(1-6) linked glucose units to generate branched long chain glycogen. GS activity is controlled via ubiquitination by the Laforin–Malin complex, maintaining soluble glycogen. Simultaneously, the LUBAC ubiquitinates glycogen residues for glycophagy targeting, releasing free glucosamine and glucose units. The hexosamine pathway and glycogenesis pathway channel these precursors to synthesis nucleotide-activated sugars which are required for N-glycan synthesis. Created in BioRender. Mitra, S. (2026) https://BioRender.com/wxgj6xj (accessed on 7 May 2026).

To conclude, our review suggests that glycogen metabolism, glycosylation, and ubiquitination are all important cellular functions somehow intertwined to control the healthy nature of our cells. A schematic of this connection is represented in Figure 3. Until recently, they have been studied in individual contexts. However, the inter-dependency of these three pathways has just started emerging and needs extensive research in the field. This will help to open up new possibilities of the treatment of devastating diseases imparted by the aberrant mechanisms involved in them.

## Figures and Tables

**Table 1:** Glycogen storage disorders with aberrant Glycosylation.

GSD Subtype	Gene	Glycosylation Altered
GSD type Ia	*G6PC*	Increased N-glycan branching, reduced fucosylation [58].
GSD type Ib	*SLC37A4*	Reduced N-glycan branching, increased fucosylation, serum transferrin hypoglycosylation [58,59].
GSD type III	*AGL*	Serum apolipoprotein C-III hyposialylation and increased aglycosylated form [60], reduced GlcNAc derived from glycans and reduced free UDP-GlcNAc [26,60].
GSD type IX	*PHK*	Reduced serum apolipoprotein glycosylation [60].
GSD type XIV	*PGM1*	Protein hypoglycosylation and reduced UDP-Galactose levels [60].
Lafora disease	*EPM2A, NHLRC1*	Reduced N-glycosylation and reduced free UDP-GlcNAc [26].
Severe congenital neutropenia	*G6PC3*	Truncated N and O glycosylation in neutrophils [61].

**Table 2:** Therapeutic strategies for treating CDGs and GSDs.

CDG/GSD Subtype	Therapeutic Strategy	Mode of Action
MPI-CDG	Monosaccharide supplementation: Oral D-Mannose administration	Providing substrate directly to the downstream enzymes of the pathway synthesizing Mannose-6-phosphate and GDP-Mannose [79].
PGM1-CDG, SLC35A2-CDG	Monosaccharide supplementation: Oral D-Galactose administration	Increases intracellular pools of UDP-Galactose [80].
SLC35C1-CDG	Monosaccharide supplementation: Oral L-Fucose administration	Increases intracellular concentrations of GDP-Fucose [77].
9SLC39A8-CDG, CAD-CDG	Cofactor supplementation therapy: Oral supplementation of manganese or uridine	Manganese acts as a cofactor and supplementation helps in stabilizing mutant enzymes. Uridine supplementation elevates nucleotide sugar substrates for glycosyltransferases [97].
PMM2-CDG	Small molecule chaperons: Acetazolamide	Acts as a chaperone and improves cerebral clinical symptoms [98].
GSD Ia	AAV-mediated gene therapy	Rescues expression of glucose-6-phosphatase (murine, canine) [57].
		AAV8-mediated rescue of glucose-6-phosphatase catalytic subunit (human) [89].
GSD Ib	AAV-mediated gene therapy	Rescues expression of glucose-6-phosphate transporter (murine) [57].
GSD II (Pompe disease)	Enzyme replacement therapy	Patients (>18 years old) infused with recombinant human (rh) acid α-glucosidase (GAA) every other week (human) [99].
		Intravenous delivery of rhGAA with mannose-6-phosphate moieties that facilitate uptake into the cell and lysosome (human). Approved for use in the US as Lumizyme and Nexviazyme [93,94].
	AAV-mediated gene therapy	AAV8-mediated rescue of expression of GAA (murine) [100].
GSD III	AAV-mediated gene therapy	AAV-mediated overexpression of GAA in liver and dual AAV-mediated expression of glycogen debranching enzyme (GDE) (murine) [101].
		Two AAV vectors with expression of bacterial GDE, one specifically targeted to liver (murine) [102].
GSD IV	AAV-mediated gene therapy	AAV9-mediated rescue of expression of glycogen branching enzyme [103].
GSD V (McCardle disease)	AAV-mediated gene therapy	AAV8-mediated rescue of expression of muscle glycogen phosphorylase (PYGM) (murine) [104].
		AAV2/AAV5-mediated rescue of expression of PYGM (ovine) [105].
	Protein injection therapy	Intramuscular injection of notexin (myotoxic snake venom protein) causes muscle tissue damage and subsequent re-expression of a non-PYGM, non-mutated isoform of glycogen phosphorylase (ovine) [106].
Lafora disease	Small molecule therapy: oral Metformin	Hypothesized to decrease glycogen levels through inhibition of mitochondrial Gpd2, and activation of AMPK with subsequent deactivation of GS [107].

## Data Availability

No new data were created or analyzed in this study. Data sharing is not applicable.
